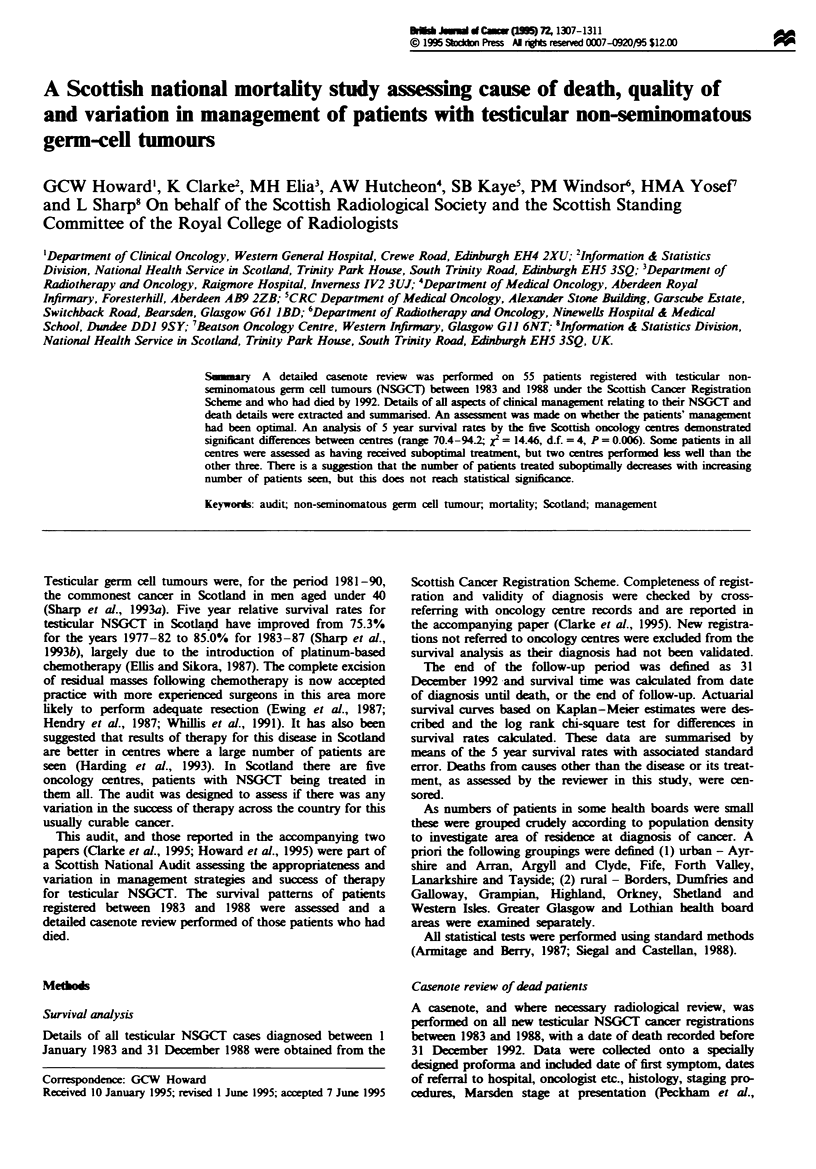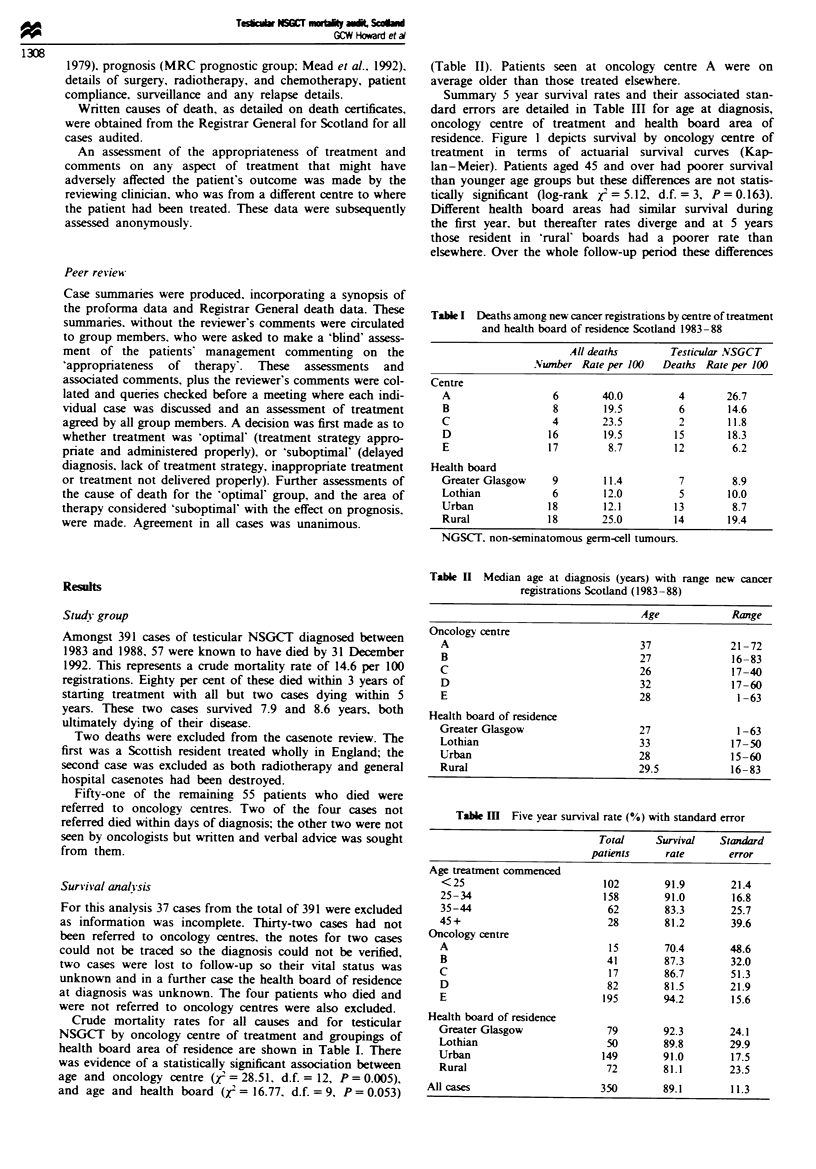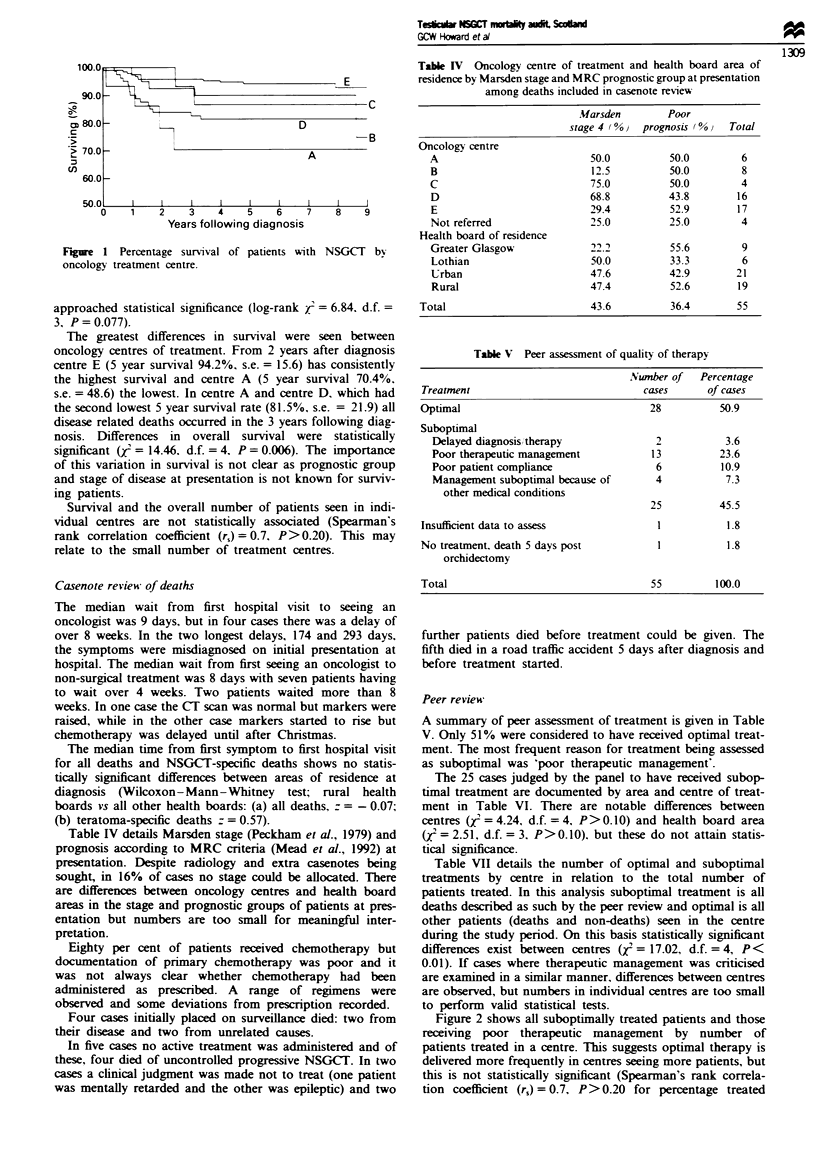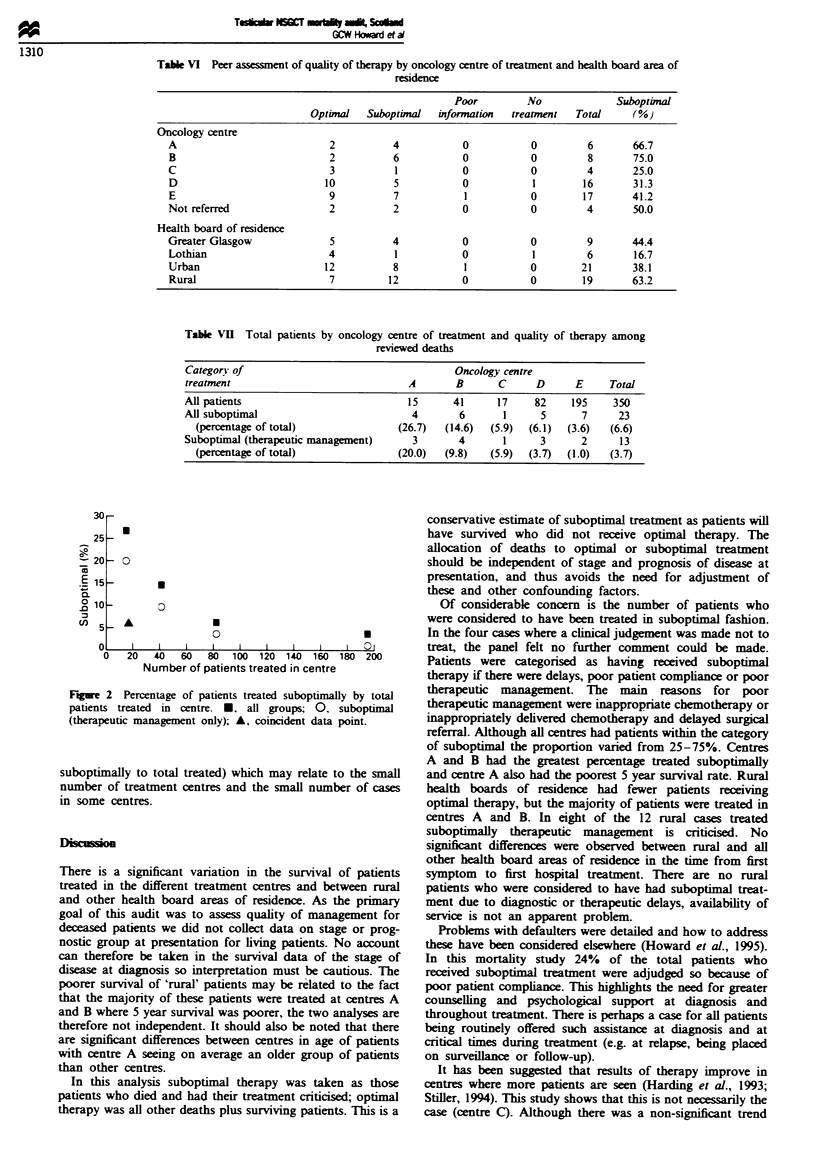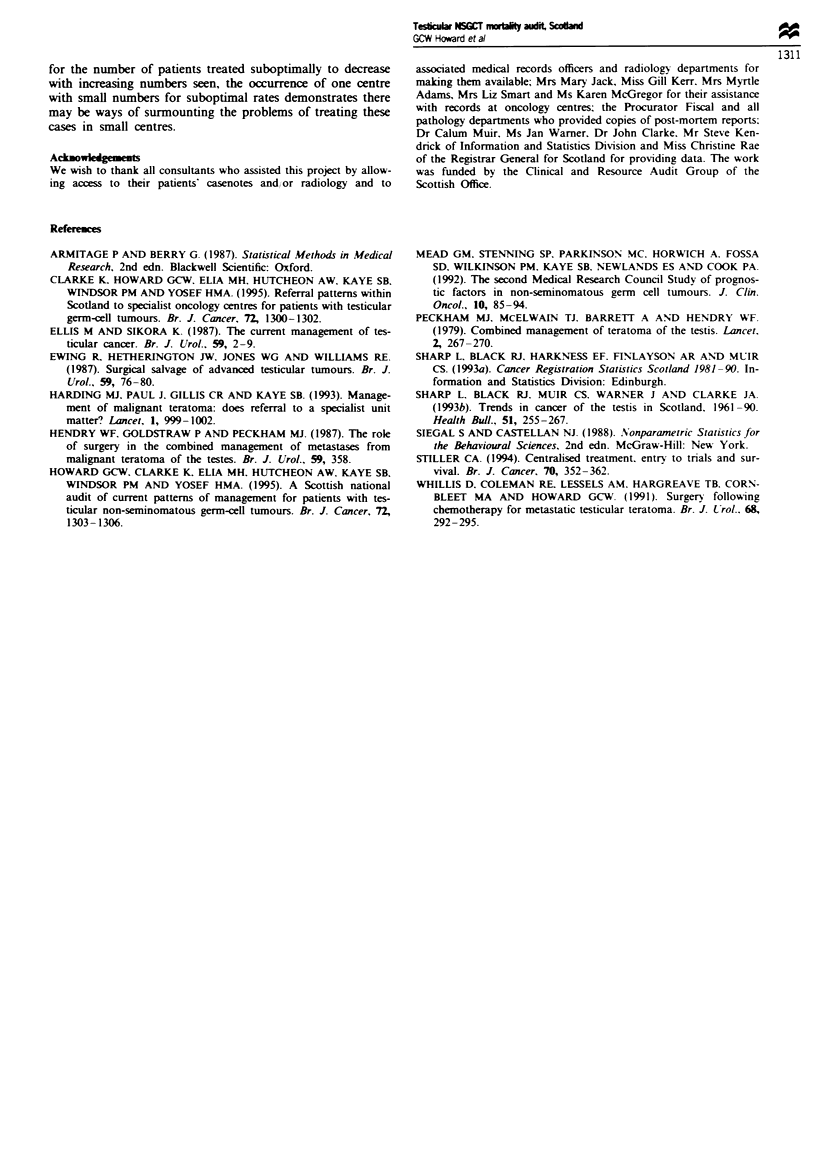# A Scottish national mortality study assessing cause of death, quality of and variation in management of patients with testicular non-seminomatous germ-cell tumours. The Scottish Radiological Society and the Scottish Standing Committee of the Royal College of Radiologists.

**DOI:** 10.1038/bjc.1995.506

**Published:** 1995-11

**Authors:** G. C. Howard, K. Clarke, M. H. Elia, A. W. Hutcheon, S. B. Kaye, P. M. Windsor, H. M. Yosef, L. Sharp

**Affiliations:** Department of Clinical Oncology, Western General Hospital, Edinburgh, UK.

## Abstract

A detailed casenote review was performed on 55 patients registered with testicular non-seminomatous germ cell tumours (NSGCT) between 1983 and 1988 under the Scottish Cancer Registration Scheme and who had died by 1992. Details of all aspects of clinical management relating to their NSGCT and death details were extracted and summarised. An assessment was made on whether the patients' management had been optimal. An analysis of 5 year survival rates by the five Scottish oncology centres demonstrated significant differences between centres (range 70.4-94.2; chi 2 = 14.46, d.f. = 4, P = 0.006). Some patients in all centres were assessed as having received suboptimal treatment, but two centres performed less well than the other three. There is a suggestion that the number of patients treated suboptimally decreases with increasing number of patients seen, but this does not reach statistical significance.


© 1995 Stockton Press All rights reserved 0007-0920/95 $12.00

### A Scottish national mortality study assessing cause of death, quality of and variation in management of patients with testicular non-seminomatous germ-cell tumours

GCW Howard[1], K Clarke[2], MH Elia[3], AW Hutcheon[4], SB Kaye[5], PM Windsor[6], HMA Yosef[7] and L Sharp[8] On behalf of the Scottish Radiological Society and the Scottish Standing Committee of the Royal College of Radiologists

[1]*Department of Clinical Oncology, Western General Hospital, Crewe Road, Edinburgh EH4 2XU;* [2]*Information & Statistics Division, National Health Service in Scotland, Trinity Park House, South Trinity Road, Edinburgh EH5 3SQ;* [3]*Department of Radiotherapy and Oncology, Raigmore Hospital, Inverness IV2 3UJ;* [4]*Department of Medical Oncology, Aberdeen Royal Infirmary, Foresterhill, Aberdeen AB9 2ZB;* [5]*CRC Department of Medical Oncology, Alexander Stone Building, Garscube Estate, Switchback Road, Bearsden, Glasgow G61 1BD;* [6]*Department of Radiotherapy and Oncology, Ninewells Hospital & Medical School, Dundee DD1 9SY;* [7]*Beatson Oncology Centre, Western Infirmary, Glasgow G11 6NT;* [8]*Information & Statistics Division, National Health Service in Scotland, Trinity Park House, South Trinity Road, Edinburgh EH5 3SQ, UK.*

**Summary** A detailed casenote review was performed on 55 patients registered with testicular non-seminomatous germ cell tumours (NSGCT) between 1983 and 1988 under the Scottish Cancer Registration Scheme and who had died by 1992. Details of all aspects of clinical management relating to their NSGCT and death details were extracted and summarised. An assessment was made on whether the patients' management had been optimal. An analysis of 5 year survival rates by the five Scottish oncology centres demonstrated significant differences between centres (range 70.4–94.2; $\chi^2 = 14.46$, d.f. = 4, $P = 0.006$). Some patients in all centres were assessed as having received suboptimal treatment, but two centres performed less well than the other three. There is a suggestion that the number of patients treated suboptimally decreases with increasing number of patients seen, but this does not reach statistical significance.

**Keywords**: audit; non-seminomatous germ cell tumour; mortality; Scotland; management

Testicular germ cell tumours were, for the period 1981–90, the commonest cancer in Scotland in men aged under 40 (Sharp *et al.*, 1993*a*). Five year relative survival rates for testicular NSGCT in Scotland have improved from 75.3% for the years 1977–82 to 85.0% for 1983–87 (Sharp *et al.*, 1993*b*), largely due to the introduction of platinum-based chemotherapy (Ellis and Sikora, 1987). The complete excision of residual masses following chemotherapy is now accepted practice with more experienced surgeons in this area more likely to perform adequate resection (Ewing *et al.*, 1987; Hendry *et al.*, 1987; Whillis *et al.*, 1991). It has also been suggested that results of therapy for this disease in Scotland are better in centres where a large number of patients are seen (Harding *et al.*, 1993). In Scotland there are five oncology centres, patients with NSGCT being treated in them all. The audit was designed to assess if there was any variation in the success of therapy across the country for this usually curable cancer.

This audit, and those reported in the accompanying two papers (Clarke *et al.*, 1995; Howard *et al.*, 1995) were part of a Scottish National Audit assessing the appropriateness and variation in management strategies and success of therapy for testicular NSGCT. The survival patterns of patients registered between 1983 and 1988 were assessed and a detailed casenote review performed of those patients who had died.

### Methods

### *Survival analysis*

Details of all testicular NSGCT cases diagnosed between 1 January 1983 and 31 December 1988 were obtained from the Scottish Cancer Registration Scheme. Completeness of registration and validity of diagnosis were checked by cross-referring with oncology centre records and are reported in the accompanying paper (Clarke *et al.*, 1995). New registrations not referred to oncology centres were excluded from the survival analysis as their diagnosis had not been validated.

The end of the follow-up period was defined as 31 December 1992 and survival time was calculated from date of diagnosis until death, or the end of follow-up. Actuarial survival curves based on Kaplan–Meier estimates were described and the log rank chi-square test for differences in survival rates calculated. These data are summarised by means of the 5 year survival rates with associated standard error. Deaths from causes other than the disease or its treatment, as assessed by the reviewer in this study, were censored.

As numbers of patients in some health boards were small these were grouped crudely according to population density to investigate area of residence at diagnosis of cancer. A priori the following groupings were defined (1) urban – Ayrshire and Arran, Argyll and Clyde, Fife, Forth Valley, Lanarkshire and Tayside; (2) rural – Borders, Dumfries and Galloway, Grampian, Highland, Orkney, Shetland and Western Isles. Greater Glasgow and Lothian health board areas were examined separately.

All statistical tests were performed using standard methods (Armitage and Berry, 1987; Siegal and Castellan, 1988).

### *Casenote review of dead patients*

A casenote, and where necessary radiological review, was performed on all new testicular NSGCT cancer registrations between 1983 and 1988, with a date of death recorded before 31 December 1992. Data were collected onto a specially designed proforma and included date of first symptom, dates of referral to hospital, oncologist etc., histology, staging procedures, Marsden stage at presentation (Peckham *et al.*,

Correspondence: GCW Howard
Received 10 January 1995; revised 1 June 1995; accepted 7 June 1995

1979), prognosis (MRC prognostic group; Mead *et al.*, 1992), details of surgery, radiotherapy, and chemotherapy, patient compliance, surveillance and any relapse details.

Written causes of death, as detailed on death certificates, were obtained from the Registrar General for Scotland for all cases audited.

An assessment of the appropriateness of treatment and comments on any aspect of treatment that might have adversely affected the patient's outcome was made by the reviewing clinician, who was from a different centre to where the patient had been treated. These data were subsequently assessed anonymously.

*Peer review*

Case summaries were produced, incorporating a synopsis of the proforma data and Registrar General death data. These summaries, without the reviewer's comments were circulated to group members, who were asked to make a 'blind' assessment of the patients' management commenting on the 'appropriateness of therapy'. These assessments and associated comments, plus the reviewer's comments were collated and queries checked before a meeting where each individual case was discussed and an assessment of treatment agreed by all group members. A decision was first made as to whether treatment was 'optimal' (treatment strategy appropriate and administered properly), or 'suboptimal' (delayed diagnosis, lack of treatment strategy, inappropriate treatment or treatment not delivered properly). Further assessments of the cause of death for the 'optimal' group, and the area of therapy considered 'suboptimal' with the effect on prognosis, were made. Agreement in all cases was unanimous.

### Results

*Study group*

Amongst 391 cases of testicular NSGCT diagnosed between 1983 and 1988, 57 were known to have died by 31 December 1992. This represents a crude mortality rate of 14.6 per 100 registrations. Eighty per cent of these died within 3 years of starting treatment with all but two cases dying within 5 years. These two cases survived 7.9 and 8.6 years, both ultimately dying of their disease.

Two deaths were excluded from the casenote review. The first was a Scottish resident treated wholly in England; the second case was excluded as both radiotherapy and general hospital casenotes had been destroyed.

Fifty-one of the remaining 55 patients who died were referred to oncology centres. Two of the four cases not referred died within days of diagnosis; the other two were not seen by oncologists but written and verbal advice was sought from them.

*Survival analysis*

For this analysis 37 cases from the total of 391 were excluded as information was incomplete. Thirty-two cases had not been referred to oncology centres, the notes for two cases could not be traced so the diagnosis could not be verified, two cases were lost to follow-up so their vital status was unknown and in a further case the health board of residence at diagnosis was unknown. The four patients who died and were not referred to oncology centres were also excluded.

Crude mortality rates for all causes and for testicular NSGCT by oncology centre of treatment and groupings of health board area of residence are shown in Table I. There was evidence of a statistically significant association between age and oncology centre ($\chi^2 = 28.51$, d.f. = 12, $P = 0.005$), and age and health board ($\chi^2 = 16.77$, d.f. = 9, $P = 0.053$) (Table II). Patients seen at oncology centre A were on average older than those treated elsewhere.

Summary 5 year survival rates and their associated standard errors are detailed in Table III for age at diagnosis, oncology centre of treatment and health board area of residence. Figure 1 depicts survival by oncology centre of treatment in terms of actuarial survival curves (Kaplan–Meier). Patients aged 45 and over had poorer survival than younger age groups but these differences are not statistically significant (log-rank $\chi^2 = 5.12$, d.f. = 3, $P = 0.163$). Different health board areas had similar survival during the first year, but thereafter rates diverge and at 5 years those resident in 'rural' boards had a poorer rate than elsewhere. Over the whole follow-up period these differences

**Table I** Deaths among new cancer registrations by centre of treatment and health board of residence Scotland 1983–88

| | *All deaths* | | *Testicular NSGCT* | |
|---|---|---|---|---|
| | *Number* | *Rate per 100* | *Deaths* | *Rate per 100* |
| Centre | | | | |
| A | 6 | 40.0 | 4 | 26.7 |
| B | 8 | 19.5 | 6 | 14.6 |
| C | 4 | 23.5 | 2 | 11.8 |
| D | 16 | 19.5 | 15 | 18.3 |
| E | 17 | 8.7 | 12 | 6.2 |
| Health board | | | | |
| Greater Glasgow | 9 | 11.4 | 7 | 8.9 |
| Lothian | 6 | 12.0 | 5 | 10.0 |
| Urban | 18 | 12.1 | 13 | 8.7 |
| Rural | 18 | 25.0 | 14 | 19.4 |

NGSCT, non-seminatomous germ-cell tumours.

**Table II** Median age at diagnosis (years) with range new cancer registrations Scotland (1983–88)

| | *Age* | *Range* |
|---|---|---|
| Oncology centre | | |
| A | 37 | 21–72 |
| B | 27 | 16–83 |
| C | 26 | 17–40 |
| D | 32 | 17–60 |
| E | 28 | 1–63 |
| Health board of residence | | |
| Greater Glasgow | 27 | 1–63 |
| Lothian | 33 | 17–50 |
| Urban | 28 | 15–60 |
| Rural | 29.5 | 16–83 |

**Table III** Five year survival rate (%) with standard error

| | *Total patients* | *Survival rate* | *Standard error* |
|---|---|---|---|
| Age treatment commenced | | | |
| <25 | 102 | 91.9 | 21.4 |
| 25–34 | 158 | 91.0 | 16.8 |
| 35–44 | 62 | 83.3 | 25.7 |
| 45+ | 28 | 81.2 | 39.6 |
| Oncology centre | | | |
| A | 15 | 70.4 | 48.6 |
| B | 41 | 87.3 | 32.0 |
| C | 17 | 86.7 | 51.3 |
| D | 82 | 81.5 | 21.9 |
| E | 195 | 94.2 | 15.6 |
| Health board of residence | | | |
| Greater Glasgow | 79 | 92.3 | 24.1 |
| Lothian | 50 | 89.8 | 29.9 |
| Urban | 149 | 91.0 | 17.5 |
| Rural | 72 | 81.1 | 23.5 |
| All cases | 350 | 89.1 | 11.3 |

**Figure 1** Percentage survival of patients with NSGCT by oncology treatment centre.

approached statistical significance (log-rank $\chi^2 = 6.84$, d.f. = 3, $P = 0.077$).

The greatest differences in survival were seen between oncology centres of treatment. From 2 years after diagnosis centre E (5 year survival 94.2%, s.e. = 15.6) has consistently the highest survival and centre A (5 year survival 70.4%, s.e. = 48.6) the lowest. In centre A and centre D, which had the second lowest 5 year survival rate (81.5%, s.e. = 21.9) all disease related deaths occurred in the 3 years following diagnosis. Differences in overall survival were statistically significant ($\chi^2 = 14.46$, d.f. = 4, $P = 0.006$). The importance of this variation in survival is not clear as prognostic group and stage of disease at presentation is not known for surviving patients.

Survival and the overall number of patients seen in individual centres are not statistically associated (Spearman's rank correlation coefficient $(r_s) = 0.7$, $P > 0.20$). This may relate to the small number of treatment centres.

*Casenote review of deaths*

The median wait from first hospital visit to seeing an oncologist was 9 days, but in four cases there was a delay of over 8 weeks. In the two longest delays, 174 and 293 days, the symptoms were misdiagnosed on initial presentation at hospital. The median wait from first seeing an oncologist to non-surgical treatment was 8 days with seven patients having to wait over 4 weeks. Two patients waited more than 8 weeks. In one case the CT scan was normal but markers were raised, while in the other case markers started to rise but chemotherapy was delayed until after Christmas.

The median time from first symptom to first hospital visit for all deaths and NSGCT-specific deaths shows no statistically significant differences between areas of residence at diagnosis (Wilcoxon–Mann–Whitney test; rural health boards *vs* all other health boards: (a) all deaths, $z = -0.07$; (b) teratoma-specific deaths $z = 0.57$).

Table IV details Marsden stage (Peckham *et al.*, 1979) and prognosis according to MRC criteria (Mead *et al.*, 1992) at presentation. Despite radiology and extra casenotes being sought, in 16% of cases no stage could be allocated. There are differences between oncology centres and health board areas in the stage and prognostic groups of patients at presentation but numbers are too small for meaningful interpretation.

Eighty per cent of patients received chemotherapy but documentation of primary chemotherapy was poor and it was not always clear whether chemotherapy had been administered as prescribed. A range of regimens were observed and some deviations from prescription recorded.

Four cases initially placed on surveillance died: two from their disease and two from unrelated causes.

In five cases no active treatment was administered and of these, four died of uncontrolled progressive NSGCT. In two cases a clinical judgment was made not to treat (one patient was mentally retarded and the other was epileptic) and two further patients died before treatment could be given. The fifth died in a road traffic accident 5 days after diagnosis and before treatment started.

**Table IV** Oncology centre of treatment and health board area of residence by Marsden stage and MRC prognostic group at presentation among deaths included in casenote review

| | *Marsden stage 4 (%)* | *Poor prognosis (%)* | *Total* |
|---|---|---|---|
| Oncology centre | | | |
| A | 50.0 | 50.0 | 6 |
| B | 12.5 | 50.0 | 8 |
| C | 75.0 | 50.0 | 4 |
| D | 68.8 | 43.8 | 16 |
| E | 29.4 | 52.9 | 17 |
| Not referred | 25.0 | 25.0 | 4 |
| Health board of residence | | | |
| Greater Glasgow | 22.2 | 55.6 | 9 |
| Lothian | 50.0 | 33.3 | 6 |
| Urban | 47.6 | 42.9 | 21 |
| Rural | 47.4 | 52.6 | 19 |
| Total | 43.6 | 36.4 | 55 |

**Table V** Peer assessment of quality of therapy

| *Treatment* | *Number of cases* | *Percentage of cases* |
|---|---|---|
| Optimal | 28 | 50.9 |
| Suboptimal | | |
| Delayed diagnosis/therapy | 2 | 3.6 |
| Poor therapeutic management | 13 | 23.6 |
| Poor patient compliance | 6 | 10.9 |
| Management suboptimal because of other medical conditions | 4 | 7.3 |
| | 25 | 45.5 |
| Insufficient data to assess | 1 | 1.8 |
| No treatment, death 5 days post orchidectomy | 1 | 1.8 |
| Total | 55 | 100.0 |

*Peer review*

A summary of peer assessment of treatment is given in Table V. Only 51% were considered to have received optimal treatment. The most frequent reason for treatment being assessed as suboptimal was 'poor therapeutic management'.

The 25 cases judged by the panel to have received suboptimal treatment are documented by area and centre of treatment in Table VI. There are notable differences between centres ($\chi^2 = 4.24$, d.f. = 4, $P > 0.10$) and health board area ($\chi^2 = 2.51$, d.f. = 3, $P > 0.10$), but these do not attain statistical significance.

Table VII details the number of optimal and suboptimal treatments by centre in relation to the total number of patients treated. In this analysis suboptimal treatment is all deaths described as such by the peer review and optimal is all other patients (deaths and non-deaths) seen in the centre during the study period. On this basis statistically significant differences exist between centres ($\chi^2 = 17.02$, d.f. = 4, $P < 0.01$). If cases where therapeutic management was criticised are examined in a similar manner, differences between centres are observed, but numbers in individual centres are too small to perform valid statistical tests.

Figure 2 shows all suboptimally treated patients and those receiving poor therapeutic management by number of patients treated in a centre. This suggests optimal therapy is delivered more frequently in centres seeing more patients, but this is not statistically significant (Spearman's rank correlation coefficient $(r_s) = 0.7$, $P > 0.20$ for percentage treated

**Table VI** Peer assessment of quality of therapy by oncology centre of treatment and health board area of residence

| | Optimal | Suboptimal | Poor information | No treatment | Total | Suboptimal (%) |
|---|---|---|---|---|---|---|
| Oncology centre | | | | | | |
| A | 2 | 4 | 0 | 0 | 6 | 66.7 |
| B | 2 | 6 | 0 | 0 | 8 | 75.0 |
| C | 3 | 1 | 0 | 0 | 4 | 25.0 |
| D | 10 | 5 | 0 | 1 | 16 | 31.3 |
| E | 9 | 7 | 1 | 0 | 17 | 41.2 |
| Not referred | 2 | 2 | 0 | 0 | 4 | 50.0 |
| Health board of residence | | | | | | |
| Greater Glasgow | 5 | 4 | 0 | 0 | 9 | 44.4 |
| Lothian | 4 | 1 | 0 | 1 | 6 | 16.7 |
| Urban | 12 | 8 | 1 | 0 | 21 | 38.1 |
| Rural | 7 | 12 | 0 | 0 | 19 | 63.2 |

**Table VII** Total patients by oncology centre of treatment and quality of therapy among reviewed deaths

| Category of treatment | Oncology centre A | B | C | D | E | Total |
|---|---|---|---|---|---|---|
| All patients | 15 | 41 | 17 | 82 | 195 | 350 |
| All suboptimal | 4 | 6 | 1 | 5 | 7 | 23 |
| (percentage of total) | (26.7) | (14.6) | (5.9) | (6.1) | (3.6) | (6.6) |
| Suboptimal (therapeutic management) | 3 | 4 | 1 | 3 | 2 | 13 |
| (percentage of total) | (20.0) | (9.8) | (5.9) | (3.7) | (1.0) | (3.7) |

**Figure 2** Percentage of patients treated suboptimally by total patients treated in centre. ■, all groups; O, suboptimal (therapeutic management only); ▲, coincident data point.

suboptimally to total treated) which may relate to the small number of treatment centres and the small number of cases in some centres.

### Discussion

There is a significant variation in the survival of patients treated in the different treatment centres and between rural and other health board areas of residence. As the primary goal of this audit was to assess quality of management for deceased patients we did not collect data on stage or prognostic group at presentation for living patients. No account can therefore be taken in the survival data of the stage of disease at diagnosis so interpretation must be cautious. The poorer survival of 'rural' patients may be related to the fact that the majority of these patients were treated at centres A and B where 5 year survival was poorer, the two analyses are therefore not independent. It should also be noted that there are significant differences between centres in age of patients with centre A seeing on average an older group of patients than other centres.

In this analysis suboptimal therapy was taken as those patients who died and had their treatment criticised; optimal therapy was all other deaths plus surviving patients. This is a conservative estimate of suboptimal treatment as patients will have survived who did not receive optimal therapy. The allocation of deaths to optimal or suboptimal treatment should be independent of stage and prognosis of disease at presentation, and thus avoids the need for adjustment of these and other confounding factors.

Of considerable concern is the number of patients who were considered to have been treated in suboptimal fashion. In the four cases where a clinical judgement was made not to treat, the panel felt no further comment could be made. Patients were categorised as having received suboptimal therapy if there were delays, poor patient compliance or poor therapeutic management. The main reasons for poor therapeutic management were inappropriate chemotherapy or inappropriately delivered chemotherapy and delayed surgical referral. Although all centres had patients within the category of suboptimal the proportion varied from 25–75%. Centres A and B had the greatest percentage treated suboptimally and centre A also had the poorest 5 year survival rate. Rural health boards of residence had fewer patients receiving optimal therapy, but the majority of patients were treated in centres A and B. In eight of the 12 rural cases treated suboptimally therapeutic management is criticised. No significant differences were observed between rural and all other health board areas of residence in the time from first symptom to first hospital treatment. There are no rural patients who were considered to have had suboptimal treatment due to diagnostic or therapeutic delays, availability of service is not an apparent problem.

Problems with defaulters were detailed and how to address these have been considered elsewhere (Howard *et al.*, 1995). In this mortality study 24% of the total patients who received suboptimal treatment were adjudged so because of poor patient compliance. This highlights the need for greater counselling and psychological support at diagnosis and throughout treatment. There is perhaps a case for all patients being routinely offered such assistance at diagnosis and at critical times during treatment (e.g. at relapse, being placed on surveillance or follow-up).

It has been suggested that results of therapy improve in centres where more patients are seen (Harding *et al.*, 1993; Stiller, 1994). This study shows that this is not necessarily the case (centre C). Although there was a non-significant trend for the number of patients treated suboptimally to decrease with increasing numbers seen, the occurrence of one centre with small numbers for suboptimal rates demonstrates there may be ways of surmounting the problems of treating these cases in small centres.

**Acknowledgements**
We wish to thank all consultants who assisted this project by allowing access to their patients' casenotes and/or radiology and to associated medical records officers and radiology departments for making them available; Mrs Mary Jack, Miss Gill Kerr, Mrs Myrtle Adams, Mrs Liz Smart and Ms Karen McGregor for their assistance with records at oncology centres; the Procurator Fiscal and all pathology departments who provided copies of post-mortem reports; Dr Calum Muir, Ms Jan Warner, Dr John Clarke, Mr Steve Kendrick of Information and Statistics Division and Miss Christine Rae of the Registrar General for Scotland for providing data. The work was funded by the Clinical and Resource Audit Group of the Scottish Office.

**References**

ARMITAGE P AND BERRY G. (1987). *Statistical Methods in Medical Research*, 2nd edn. Blackwell Scientific: Oxford.

CLARKE K, HOWARD GCW, ELIA MH, HUTCHEON AW, KAYE SB, WINDSOR PM AND YOSEF HMA. (1995). Referral patterns within Scotland to specialist oncology centres for patients with testicular germ-cell tumours. *Br. J. Cancer*, **72**, 1300-1302.

ELLIS M AND SIKORA K. (1987). The current management of testicular cancer. *Br. J. Urol.*, **59**, 2-9.

EWING R, HETHERINGTON JW, JONES WG AND WILLIAMS RE. (1987). Surgical salvage of advanced testicular tumours. *Br. J. Urol.*, **59**, 76-80.

HARDING MJ, PAUL J, GILLIS CR AND KAYE SB. (1993). Management of malignant teratoma: does referral to a specialist unit matter? *Lancet*, **1**, 999-1002.

HENDRY WF, GOLDSTRAW P AND PECKHAM MJ. (1987). The role of surgery in the combined management of metastases from malignant teratoma of the testes. *Br. J. Urol.*, **59**, 358.

HOWARD GCW, CLARKE K, ELIA MH, HUTCHEON AW, KAYE SB, WINDSOR PM AND YOSEF HMA. (1995). A Scottish national audit of current patterns of management for patients with testicular non-seminomatous germ-cell tumours. *Br. J. Cancer*, **72**, 1303-1306.

MEAD GM, STENNING SP, PARKINSON MC, HORWICH A, FOSSA SD, WILKINSON PM, KAYE SB, NEWLANDS ES AND COOK PA. (1992). The second Medical Research Council Study of prognostic factors in non-seminomatous germ cell tumours. *J. Clin. Oncol.*, **10**, 85-94.

PECKHAM MJ, MCELWAIN TJ, BARRETT A AND HENDRY WF. (1979). Combined management of teratoma of the testis. *Lancet*, **2**, 267-270.

SHARP L, BLACK RJ, HARKNESS EF, FINLAYSON AR AND MUIR CS. (1993*a*). *Cancer Registration Statistics Scotland 1981-90*. Information and Statistics Division: Edinburgh.

SHARP L, BLACK RJ, MUIR CS, WARNER J AND CLARKE JA. (1993*b*). Trends in cancer of the testis in Scotland, 1961-90. *Health Bull.*, **51**, 255-267.

SIEGAL S AND CASTELLAN NJ. (1988). *Nonparametric Statistics for the Behavioural Sciences*, 2nd edn. McGraw-Hill: New York.

STILLER CA. (1994). Centralised treatment, entry to trials and survival. *Br. J. Cancer*, **70**, 352-362.

WHILLIS D, COLEMAN RE, LESSELS AM, HARGREAVE TB, CORNBLEET MA AND HOWARD GCW. (1991). Surgery following chemotherapy for metastatic testicular teratoma. *Br. J. Urol.*, **68**, 292-295.